# Building faculty capacity for competency-based midwifery education in Rwanda - a cross-sectional study

**DOI:** 10.1186/s12909-025-08034-5

**Published:** 2025-10-02

**Authors:** Malin Bogren, Menelas Nkeshimana, Frida Temple, Marie Claire Iryanyawera, Darlene Ineza, Innocent Nzabahimana, Peninah Ingabire, Jean de Dieu Uwimana, Renata Tallarico, Olugbemiga Adelakin, Kerstin Erlandsson

**Affiliations:** 1 Institute of Health and Care Sciences, Sahlgrenska Academy, University of Gothenburg, Arvid Wallgrens backe, House 1, Gothenburg, 413 46 Sweden; 2 Health Workforce Development Department, Ministry of Health, Kigali, Rwanda; 3 United Nations Population Fund, Kigali, Rwanda; 4 Directorate of Teaching and Learning Enhancement, University of Rwanda, Kigali, Rwanda; 5 School of Health and Welfare, Dalarna University, Falun, Sweden

**Keywords:** Midwifery faculty, Midwifery education, Competency-based education, Survey, East africa

## Abstract

**Background:**

Competency-based education is globally recognized as the standard for preparing midwives to provide high-quality, evidence-based care. In 2024, Rwanda introduced a standardized, competency-based curriculum for midwifery education aligned with the International Confederation of Midwives (ICM) Essential Competencies. However, the successful implementation of this curriculum depends on the capacity of midwifery educators to deliver it effectively. Understanding faculty development needs is essential for supporting this transition.

**Aim:**

To identify the specific faculty development needs of midwifery educators in Rwanda to inform strategies for strengthening their capacity to deliver competency-based education.

**Method:**

A cross-sectional mixed-methods study was conducted in December 2024 using an anonymous online survey. The survey was conducted immediately following the curriculum introduction. Midwifery faculty from all eight institutions offering midwifery education in Rwanda were invited to participate. The survey included Likert-scale and open-ended questions to assess training needs across various educational domains. Quantitative data were analysed using descriptive statistics, and qualitative responses were textually analysed.

**Results:**

A total of 48 out of 60 midwifery educators responded. The highest reported needs were in curriculum design and module development (79%), scientific writing (79%), management and leadership (79%), and research skills (77%). Capacity needs were also noted in clinical teaching, particularly using simulation (66%), and in the clinical environment (77%). Faculty expressed preferences for faculty development programs using blended learning with predominantly face-to-face components, and they favored intensive, short-term training formats.

**Conclusion:**

Midwifery faculty in Rwanda expressed strong needs for capacity development across education, leadership, and research domains. These findings highlight the importance of targeted, context-specific faculty development initiatives. Given the shared challenges across low-resource settings, the findings may be transferable to similar contexts aiming to develop faculty development programs aligned with global standards.

## Introduction

Ensuring high-quality midwifery education requires a well-prepared faculty capable of educating the next generation of midwives [1]. Midwives play a crucial role in improving maternal and neonatal health outcomes and advancing Sustainable Development Goal 3 on health [2]. The quality of midwifery education directly influences the ability of midwives to provide safe, effective care, which in turn depends on educators’ preparedness to deliver competency-based education [3–5]. Midwifery educators must possess not only clinical expertise but also strong pedagogical skills, leadership abilities, and proficiency in competency-based assessments [6, 7]. Faculty development is therefore essential to equipping educators with the skills needed to implement evidence-based midwifery education [8]. Strengthening faculty capacity enhances the quality of education programs, ultimately improving maternity care and reducing maternal and newborn mortality [9]. However, challenges persist in implementing and sustaining competency-based education, particularly in low-resource settings [5]. Understanding faculty development needs is therefore crucial for designing targeted interventions that support the effective integration of competency-based education and ensure long-term improvements in midwifery education and practice [10].

Competency-based education is now the global standard for educating healthcare professionals, including midwives, to ensure graduates develop the competencies required for safe, high-quality maternal and newborn care [11, 12]. Unlike traditional, knowledge-based models, competency-based education emphasizes learner-centered, practice-oriented approaches that focus on measurable competencies in critical thinking, clinical decision making, communication, and collaboration [13]. The International Confederation of Midwives (ICM) defines Essential Competencies for Midwifery Practice, outlining the knowledge, skills, and ethical values required for professional behavior. These competencies were updated in 2024 to align with evolving healthcare demands, ensuring midwives are adequately prepared to provide high-quality reproductive and perinatal care [12]. The successful implementation of competency-based education depends on well-trained faculty who can translate the full range of essential midwifery competencies into effective teaching and assessment strategies [12, 13].

However, a lack of faculty training and institutional support has hindered the integration of competency-based education in midwifery programs across Africa [14]. A scoping review highlighted difficulties in sustaining competency-based education due to inadequate faculty support and weak monitoring systems [5]. Without well-prepared educators, the transition to competency-based education remains fragmented, limiting its potential to enhance midwifery education and improve maternal and neonatal health outcomes [10].

In 2024, Rwanda introduced a standardized, competency-based curriculum for its Diploma and Bachelor of Midwifery programs, aligning them with ICM and national standards [15]. However, its success relies on educators who can effectively implement competency-based education. Hence, this study aims to identify the specific faculty development needs of midwifery educators in Rwanda and propose evidence-based strategies to strengthen faculty capacity. By addressing these needs, midwifery programs can better prepare educators to train future midwives who can meet the evolving healthcare demands of mothers and newborns.

## Method

### Study design

This study employed a cross-sectional design with a mixed-methods approach [16]. Midwifery faculty from the eight midwifery programs in Rwanda participated. Data were collected through an online survey. The survey combined closed-ended items, including a 10-point Likert scale, with optional open-text responses. This allowed for both quantitative measurement of faculty training needs across key areas (e.g., curriculum design, simulation, clinical teaching, research, leadership) and qualitative elaboration to capture contextual nuances and practical challenges. The integration of these complementary data sources provided both breadth and depth, thereby justifying a mixed-methods approach. The study received ethical approval from the Rwanda National Ethics Committee (ID: RNEC 591/24).

### Setting

Rwanda has embarked on a health sector initiative called the 4 × 4 reform [17], aimed at quadrupling the healthcare workforce within four years, while also enhancing the quality of healthcare education provision. Midwifery education is a core priority in the reform. At the time of the survey, the country had eight institutions offering midwifery education, including:

1. Adventist University of Central Africa
2. Africa Health Sciences University
3. East African Christian College
4. Institut Catholique de Kabgayi
5. Institut Superieur de Ruhengeli
6. Kibogora Polytechnic
7. Ruli Higher Institute of Health
8. University of Rwanda-College of Medicine and Health Sciences

Together, these institutions train around 2600 students annually and are supported by approximately 60 core midwifery faculty members. Recognizing the need for high-quality, standardized midwifery education, the government has invested in curriculum reform and faculty development in collaboration with key stakeholders. In response, the Ministry of Health with the support of United Nations Population Fund (UNFPA), has standardized and culturally adapted the midwifery curricula at the diploma and bachelor levels, aligning with the ICM’s Essential Competencies for Midwifery Practice [12]. Before this reform, each institution followed its own midwifery curriculum, resulting in large variations in course content and clinical hours, sometimes differing by up to 900 h. The inclusion of numerous non-midwifery subjects further diluted focus on essential midwifery competencies. The new standardized, competency-based curriculum addresses these gaps by setting national benchmarks for core midwifery subjects and minimum hours, while still allowing institutions flexibility to add complementary content.

With these competency-based curricula now in place, the next phase of this reform focuses on assessing faculty needs to ensure their effective implementation and sustainability [15]. At the time of the study, the curricula had not yet been rolled out nationally; instead, implementation is planned in phases, aligned with faculty development training that is being designed based on identified needs.

### Recruitment, sampling, and data collection

To identify the faculty development needs for delivering competency-based midwifery education in Rwanda, we employed a purposive convenience sampling strategy to invite faculty members—those holding an official faculty title—from institutions where midwifery is taught as a stand-alone program and where their responsibilities include teaching core midwifery subjects. Faculty from institutions preparing to launch midwifery programs were also included to ensure broad representation across different educational settings. This approach allowed participation from faculty at all eight midwifery education institutions while excluding staff midwives at teaching hospitals and clinical supervisors. In the Rwandan context, midwifery educators also serve as clinical supervisors. However, ward-based clinical supervisors without teaching responsibilities at higher education institutions were excluded, as they do not hold an official faculty title.

Recruitment for this online survey took place in December 2024 through the Ministry of Health and the Heads of Departments at the respective institutions. Participants received an email invitation from their department head, which included details about the study. The researchers worked closely with the institutional leadership to encourage participation and maximize response rates.

Data were collected via an anonymous online survey administered via Google Form. Participants accessed the survey through a generic link provided on recruitment flyers attached to the email invitation, including information about their participation and how data would be used, and consent was implied by completing the survey. We used a tested survey tool originally developed for a study in the Asia-Pacific region [18] and adapted it to the Rwandan context through expert review by national researchers. Wording and terminology were adjusted for cultural and institutional relevance, and additional items were included to reflect national priorities such as the adoption of competency-based curricula. The Rwandan survey comprised 28 multiple-choice questions, some with possibilities to provide several response alternatives to the same question, with optional comment sections, allowing faculty to self-assess their training needs in delivering competency-based midwifery education aligned with ICM’s Essential Competencies for Midwifery Practice [11]. To assess faculty perceptions of their training needs across various areas of midwifery education, the survey incorporated a 10-point Likert scale, ranging from *strongly agree* to *strongly disagree*. In addition to multiple-choice and Likert-scale questions, the survey included optional comment boxes where participants could elaborate on their experiences and perspectives in their own words. These open-text responses provided qualitative data to complement the quantitative findings. The survey took approximately 15 min to complete. See Appendix 1 for the full survey.

### Data analysis

The responses in Google Form were analysed using Microsoft Excel for quantitative descriptive statistics including frequency and percentages [16]. Textual data from the comment boxes were analysed using Malterud’s systematic text condensation [19]. This analysis involved four structured steps: [1] reading all responses to gain an overall impression and identify preliminary themes [2], identifying and coding meaning units related to faculty development needs [3], condensing the content of each code group while preserving the core meaning, and [4] synthesizing the condensates into thematic descriptions that complemented the quantitative findings, illustrated with representative quotes. For the Likert-scale data on training needs, responses indicating a high perceived need (scores from 6 to 10 on the 10-point scale) were combined and reported as percentages for each area (face-to-face teaching, blended/online teaching, clinical teaching using simulation, clinical teaching in the clinical environment, curriculum design/program and module development, conduct of research, writing manuscripts for publication, management and leadership). The cutoff of 6–10 was selected because the midpoint (five) was considered neutral, and values above this indicate an affirmative perception of need. Grouping the upper range also allowed for clearer descriptive reporting while preserving sufficient sample size for meaningful interpretation.

## Results

Forty-eight midwifery faculty completed the survey. The vast majority 77.1% (*n* = 37) were female, and 22.9% (*n* = 11) were male. On average, the years of experience as a midwifery faculty member was 7.2 years, with a range of 1–26 years of experience. Regarding educational qualifications, 68.8% (*n* = 33) held a master’s degree, 20.8% (*n* = 10) held a bachelor’s degree, 8.3% (*n* = 4) held a PhD, and 2.1% (*n* = 1) was a PhD candidate.

### Faculty capacity strengthening

The top three identified areas for faculty development, all with 79% positive responses, were Management and leadership, Scientific manuscript writing, and Curriculum design and module development. The two least frequently requested areas for development were Face-to-face teaching and Clinical teaching using simulation; however, more than half of the faculty still reported a high perceived need in these areas (54% and 66%, respectively).

In addition to the aspects of faculty practice listed in the survey questions, the free-text comments associated with faculty capacity strengthening identified a few additional ones. Respondents identified the need for strengthening capacity in artificial intelligence in teaching and learning, grant writing, quality improvement projects, and maternal and child health in relation to complications during labour and birth. One respondent mentioned wanting training in “… using artificial intelligence to teach, evaluate our students, and conduct research …” (Respondent 1).

### Clinical practice and simulation

Respondents were asked about their current involvement in clinical practice and the last time they worked in a clinical setting. More than half of respondents, 62% (*n* = 30), reported being actively engaged in midwifery clinical practice and that they were currently working in a clinical area of midwifery, while fewer than 37% (*n* = 18) indicated they were not. However, some of those who identified as currently practicing (62%) appeared to interpret the question as referring to their role in teaching and supervising students in clinical settings rather than doing direct clinical work. For example, one responded wrote, “I’m currently working in clinical practice with students” (Respondent 28).

When asked about the need to strengthen the capacity of clinical teaching in the clinical environment, 77% (*n* = 37) expressed a need for strengthening this area. As one respondent wrote, “I would need to strengthen my capacity in clinical teaching in the clinical environment, and I would also need to strengthen my capacity in clinical teaching using simulation” (Respondent 16).

The need for strengthening capacity in clinical teaching using simulation was expressed by 66% (*n* = 32) of the respondents. Especially mentioned areas were cardiotocography, echography, modern contraceptives including application of intrauterine devices, and obstetric maternal and newborn emergencies.

### Teaching, leadership, and research

The survey revealed several key areas where capacity development is needed among respondents in teaching, leadership, and research.

#### Face-to-face teaching

Over half of the respondents (54% *n* = 29) identified the need for capacity-building in face-to-face teaching methods, emphasizing the importance of traditional classroom-based pedagogical skills. *Blended/online teaching*: A notable 70% (*n* = 34) expressed the need for training in online and blended teaching methods, highlighting a demand for adapting to digital and hybrid learning environments. *Curriculum design*: A substantial majority (79%, *n* = 38) indicated a need for skills in curriculum design and program/module development, reflecting the importance of aligning educational content with global standards and local needs.

#### Management and leadership

Needs related to management and leadership were reported at the same level (79%, *n* = 38), demonstrating a demand for strengthening strategic and operational competencies.

#### Research and academic writing

Most respondents (77%, *n* = 37) expressed a need for capacity development in conducting research, indicating a strong interest in enhancing research skills and methodologies. A similar high percentage (79%, *n* = 38) identified the need for support in writing manuscripts for publication, underscoring the importance of academic dissemination and contribution to scientific knowledge. For example, one comment indicated a need for “Online teaching, writing manuscripts for publication, and having updates in conducting research” (Respondent 40).

### Faculty development – mode of program delivery

Faculty were asked to choose their preferred program delivery method from options including *Fully online*,* Fully face-to-face*,* Mostly online blended*, and *Mostly face-to-face blended*. The majority (77%, *n* = 37) preferred the *Mostly face-to-face blended* approach with primarily face-to-face instruction and some online activities. One respondent justified the response by commenting, “Mostly face to face with limited online because our school is located where Internet is unreliable” (Respondent 43).

**Fig. 1:**
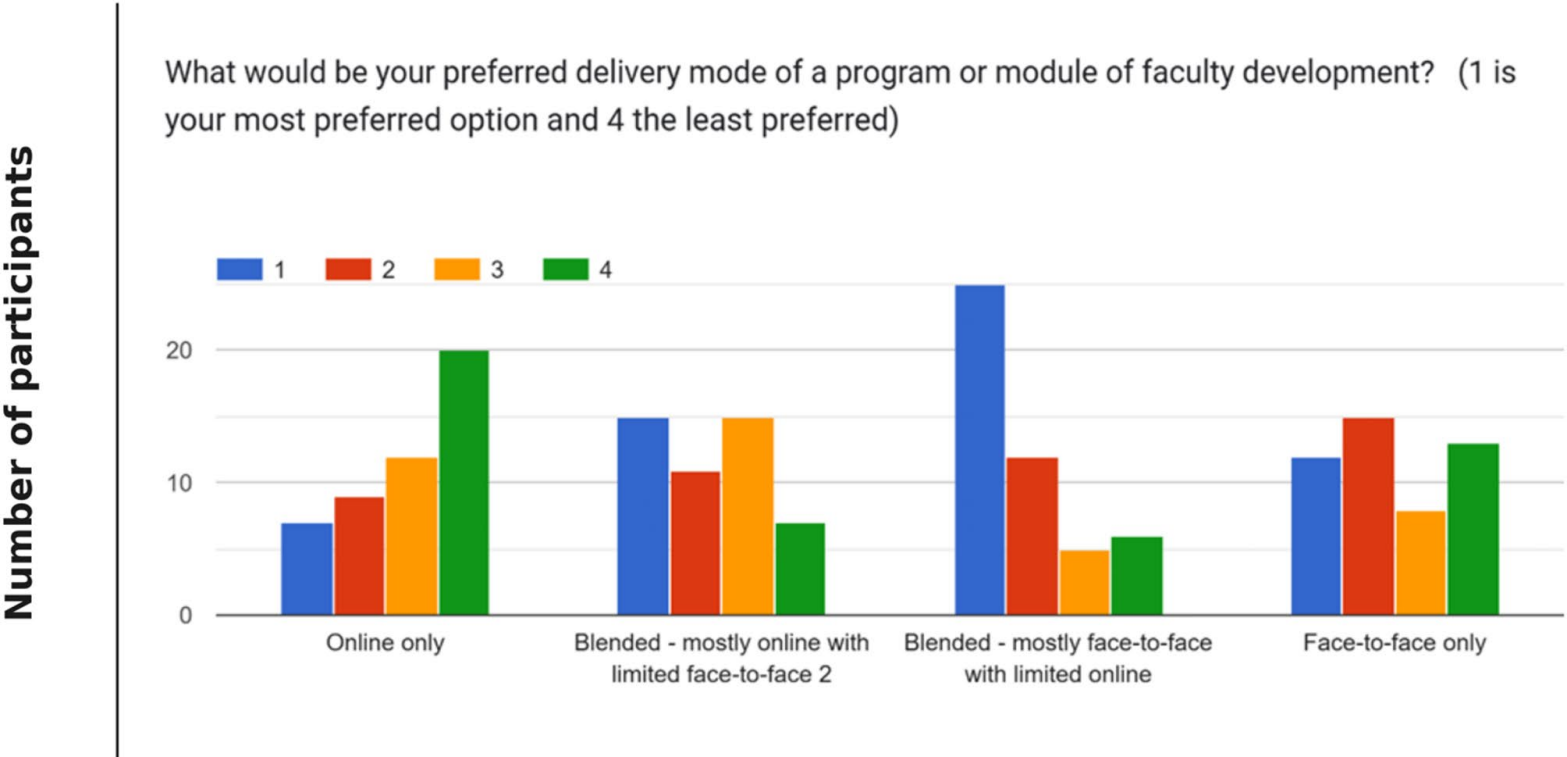
Most preferred delivery mode of a program or module of faculty development

As illustrated in Fig. 1, online only (high bar 1 and 2) was preferred by 33% (*n* = 16); blended with majority online (high bar 1 and 2) was preferred by 54% (*n* = 26); blended with majority face-to-face (high bar 1 and 2) was preferred by 77% (*n* = 37); and face-to-face only (high bar 1 and 2) was preferred by 56% (*n* = 27).

When asking the preferred format/schedule of faculty development, the options were: Single-point-in-time 10-day intensive program (over 2 weeks); One day each week for 10 weeks; Half-day every 2 weeks over a period of 6 months; and One day each month for 10 months. Respondents identified intensive program over 2 weeks as the most preferred option 69% (*n* = 33) or, alternatively, one day each week for a number of weeks 67% (*n* = 32). As one respondent stated: “A single-point-in-time intensive program can indeed be highly effective in program development, particularly in fields where deep, concentrated learning or skill acquisition is needed” (Respondent 44).

## Discussion

As part of the development of the standardized midwifery curriculum, the government of Rwanda and UNFPA commissioned this study to identify the faculty development needs of midwifery educators in Rwanda, in order to support the implementation of the newly standardized, competency-based curriculum aligned with the ICM Essential Competencies [11]. The findings reveal a strong and widespread demand for faculty development across three critical domains—education, leadership, and research—which are essential for advancing competency-based midwifery education and sustaining health system reforms like Rwanda’s 4 × 4 reform [17].

The findings from this survey identified critical needs in faculty capacity related to pedagogical methods, curriculum development, and digital competency within the context of Rwandan midwifery education. Over half of the respondents (54%) reported a need for capacity building in face-to-face teaching skills, reaffirming the ongoing relevance of traditional classroom-based pedagogy. This is particularly relevant for educators transitioning from clinical roles to academic settings, who may lack formal training in didactic methods [20].

Notably, 70% of respondents expressed a need for training in online and blended teaching methods, indicating that digital education is increasingly viewed as a vital component of health professional training. This is particularly relevant in sub-Saharan Africa, where online tools are being leveraged to enhance access, interactivity, and flexibility in learning [21, 22]. Faculty development initiatives should therefore include digital literacy and instructional design for hybrid environments, while remaining sensitive to infrastructural limitations such as inconsistent internet access, as highlighted by participant comments.

The most urgent need reported—by 79% of respondents—was in curriculum design and module development, mirroring global findings that educators often lack training in operationalizing competency-based curricula into assessable learning experiences [1, 8, 23]. As Rwanda transitions to this model, educators must be equipped to design curricula that are both globally aligned and locally responsive to maternal and neonatal health priorities.

Despite many faculty being involved in clinical teaching, 77% indicated a need to strengthen clinical teaching competencies—particularly in the use of simulation-based education and in content areas such as maternal emergencies, contraception, and diagnostic skills. This disparity suggests that clinical supervision is often conflated with structured clinical instruction, a distinction that has meaningful implications for pedagogical effectiveness and clinical competency retention [5, 11]. The growing emphasis on simulation-based education in global midwifery literature [5] supports the integration of high-fidelity simulations to bridge the theory-practice divide and foster hands-on skill development given that 50% of midwifery education takes place in clinical practice settings [24].

Leadership and management skills were identified as a major faculty development priority, with 79% of respondents highlighting this area. This finding aligns with literature that emphasizes the role of educators not only as instructors but also as institutional leaders and change agents [1]. In Rwanda’s context—where educators operate within resource-constrained environments and must navigate wide-reaching educational reforms—effective leadership is critical to institutional resilience, cross-sectoral collaboration, and evidence-informed decision making [15, 25]. This study offers valuable insights into the leadership skills required to advance midwifery education and practice. Leadership development must go beyond managerial duties to encompass professional identity formation, interprofessional collaboration, and succession planning. Strengthening leadership among early-career midwives and students, as well as formalizing distinct leadership roles within academic institutions, is vital for sustainable workforce development. These findings echo global recommendations to invest in “strong midwifery leadership” as a strategy to reduce perinatal mortality and improve maternal outcomes [26].

Moreover, academic leadership is increasingly recognized as key to faculty motivation, continuous quality improvement, and institutional visibility. Empowering educators to lead innovation, mentor peers, and represent midwifery on national and international platforms can amplify the profession’s voice in health system decision making [27]. Leadership and management training should therefore be considered a foundational element of faculty development, designed with participatory, context-sensitive approaches that draw from both local realities and international best practices.

The findings reveal a substantial demand for enhanced research capacity, with 77% of respondents indicating a need for training in research methodology, and 79% in scientific writing and publication. This underscores the dual role of midwifery educators as both teachers and knowledge producers—an essential dynamic for advancing evidence-based education and practice. Comparable studies in other low- and middle-income countries have reported similar capacity needs among midwifery faculty, often citing limited opportunities for formal training in research methodologies, data analysis, and scholarly writing [18]. Without these competencies, educators are limited in their ability to contribute to academic scholarship, inform curriculum development and clinical guidelines, and shape national health policies. Strengthening research capacity is therefore not only an academic imperative but also a policy and practice priority.

Developing these skills fosters a culture of inquiry within institutions, promotes evidence-informed teaching, and increases the visibility of midwifery education in global health dialogues. Faculty who can conduct and disseminate research are also more likely to access funding opportunities and participate in international collaborations. Faculty development programs should include structured training in research design, data analysis, ethics, and scientific communication to enable educators to generate and share rigorous, relevant knowledge that informs both pedagogy and maternal health priorities.

The faculty development needs identified in this study, closely align with the recently revised ICM Essential Competencies for Midwifery Practice [12]. This alignment underscores the timeliness of addressing these development needs to ensure that midwifery education in Rwanda not only supports the implementation of the national competency-based curricula but also remains consistent with global standards. It further highlights the importance for midwifery educators to regularly review and adapt their programs in line with evolving international competencies [12].

### Strengths and limitations

A key strength of this study is its broad institutional representation, capturing perspectives from faculty across all eight midwifery education institutions in Rwanda. This inclusivity provides valuable insight into faculty development needs at a critical time of curriculum reform and national health workforce expansion. The use of a validated and contextually adapted survey tool [18] also enhances the relevance and credibility of the findings.

However, the study has several limitations. It relied on self-reported data, which may be subject to response and recall bias. The sample size was relatively small (*n* = 48), and since the estimated number of eligible faculty was only 60, the sampling method and sample size were not determined by statistical calculations or theoretical saturation. A purposive convenience sampling strategy was used to maximize participation, which may have introduced selection bias. Furthermore, the small sample size precludes the calculation of statistical significance and limits the generalizability of the findings. The phrasing of some questions may have led to misinterpretation among respondents, and this may have affected the accuracy of some data.

Another limitation of this study is that the qualitative data were drawn only from survey comment boxes. While these responses offered useful contextual insights, they were often brief and did not allow for the depth of understanding that could be gained from more interactive qualitative methods. Future research using individual interviews or focus groups discussions would provide richer insights [16] into the specific faculty development needs and support capacity building for competency-based education. In addition, future studies should also include midwives who serve as clinical preceptors, as changes in midwifery education require that those supervising students in practice settings are equally updated and trained in the new curricula. In addition to this limitation regarding data depth, the study’s cross-sectional design provides only a snapshot at one point in time and cannot capture how needs may change as competency-based education is further implemented. Longitudinal studies would therefore be valuable [16], in tracking evolving faculty development priorities.

Despite these limitations, the study offers timely and practical evidence to inform future plans by the government of Rwanda and partners such as ICM, UNFPA and WHO regarding faculty development strategies, and to support the successful implementation of competency-based midwifery education in Rwanda.

While the study is rooted in the Rwandan context, the findings have potential relevance for pursuing the delivery of competency-based midwifery curricula in other contexts, particularly in resource-constrained environments. By adapting the online survey used in this study, other countries may develop context-specific faculty development programs aligned with global standards [28].

## Conclusion

This study provides important insights into the faculty development needs of midwifery educators in Rwanda as the country implements a competency-based curriculum aligned with international standards. Key areas identified for capacity strengthening include education, leadership, and research. These findings underscore the urgent need for structured, context-responsive faculty development programs to ensure educators are well equipped to deliver high-quality, competency-based midwifery education.

## Data Availability

The data sets used and/or analysed during the current study are available from the corresponding author upon reasonable request.
